# Pulegone Prevents Hypertension through Activation of Muscarinic Receptors and Cyclooxygenase Pathway in L-NAME-Induced Hypertensive Rats

**DOI:** 10.1155/2023/8166840

**Published:** 2023-05-09

**Authors:** Muryam Abdul Razzaq, Waqas Younis, Muhammad Nasir Hayat Malik, Tariq G. Alsahli, Alamgeer, Shah Jahan, Roma Ehsan, Arquimedes Gasparotto Junior, Asifa Bashir

**Affiliations:** ^1^Department of Pharmacology, Faculty of Pharmacy, The University of Lahore, Lahore, Pakistan; ^2^Department of Pharmacology, Physiology, and Neuroscience, New Jersey Medical School-Rutgers, Newark, NJ 07103, USA; ^3^Department of Pharmacology, College of Pharmacy, Jouf University, Sakaka, Aljouf 72341, Saudi Arabia; ^4^University College of Pharmacy, University of The Punjab, Lahore, Pakistan; ^5^Department of Immunology, University of Health Sciences, Lahore, Pakistan; ^6^Laboratory of Cardiovascular Pharmacology (LaFaC), Faculty of Health Sciences, Federal University of Grande Dourados, Dourados, MS, Brazil

## Abstract

The current study was designed to determine pulegone's antihypertensive and vasoprotective activity in L-NAME-induced hypertensive rats. Firstly, the hypotensive dose-response relationship of pulegone was evaluated in normotensive anesthetized rats using the invasive method. Secondly, the mechanism involved in hypotensive activity was determined in the presence of pharmacological drugs such as atropine/muscarinic receptor blocker (1 mg/kg), L-NAME/NOS inhibitor (20 mg/kg), and indomethacin/COX inhibitor (5 mg/kg) in anesthetized rats. Furthermore, studies were carried out to assess the preventive effect of pulegone in L-NAME-induced hypertensive rats. Hypertension was induced in rats by administering L-NAME (40 mg/kg) orally for 28 days. Rats were divided into six groups which were treated orally with tween 80 (placebo), captopril (10 mg/kg), and different doses of pulegone (20 mg/kg, 40 mg/kg, and 80 mg/kg). Blood pressure, urine volume, sodium, and body weight were monitored weekly. After 28 days, the effect of pulegone on lipid profile, hepatic markers, antioxidant enzymes, and nitric oxide was estimated from the serum of treated rats. Moreover, plasma mRNA expression of eNOS, ACE, ICAM1, and EDN1 was measured using real-time PCR. Results show that pulegone dose-dependently decreased blood pressure and heart rate in normotensive rats, with the highest effect at 30 mg/kg/i.v. The hypotensive effect of pulegone was reduced in the presence of atropine and indomethacin, whereas L-NAME did not change its hypotensive effect. Concurrent treatment with pulegone for four weeks in L-NAME-treated rats caused a reduction in both systolic blood pressure and heart rate, reversed the reduced levels of serum nitric oxide (NO), and ameliorated lipid profile and oxidative stress markers. Treatment with pulegone also improved the vascular response to acetylcholine. Plasma mRNA expression of eNOS was reduced, whereas ACE, ICAM1, and EDN1 levels were high in the L-NAME group, which was facilitated by pulegone treatment. To conclude, pulegone prevented L-NAME-induced hypertension by demonstrating a hypotensive effect through muscarinic receptors and cyclooxygenase pathway, indicating its use as a potential candidate in managing hypertension.

## 1. Introduction

Cardiovascular diseases (CVDs) are the foremost and primary cause of death and disability worldwide. Around 95% of CVD deaths are caused by ischemic heart disease (IHD), rheumatic heart disease, stroke, hypertension, myocardiopathy, and arrhythmia [1, 2]. Hypertension is a critical risk factor for the development of cardiovascular disease, which causes mortality and morbidity worldwide. It causes functional and structural abnormalities in the heart, kidneys, brain, and vasculature, which results in congestive heart failure, coronary artery disease, nephropathy, stroke, and retinopathy [3, 4]. The incidence of hypertension has been increasing globally, particularly in developing countries. By 2025, more than 500 million people are expected to be at high risk due to hypertension [5].

Muscarinic receptor activation is essential in the parasympathetic regulation of cardiovascular function. Activation of M2 muscarinic receptors reduces the contractility of atrial cardiomyocytes. It decreases heart rate by slowing the rate of spontaneous action potential firing in the sinoatrial (SA) node, thus reducing the overall cardiac output [6]. M3 receptors are mainly expressed in vascular endothelium and are involved in vasodilation by stimulating nitric oxide (NO) production, which impacts afterload and vascular resistance, thus reducing blood pressure [7]. Moreover, COX-1- and COX-2-derived prostaglandins are also essential in cardiovascular physiology. PGI2, primarily COX-2 derived, is a vasodilator expressed mainly by endothelial and vascular smooth muscle cells. It reduces blood pressure by increasing the level of cAMP, which leads to vasorelaxation [8, 9].

Modern medicine offers many classes of antihypertensive drugs, such as calcium channel blockers, ACE inhibitors, beta-blockers, and diuretics [10]. However, target blood pressures are not attained readily among patients in clinical practice [11]. Due to the frequent side effects encountered by using antihypertensive medications, researchers are using medicinal plants and phytochemicals as alternate natural remedies for treating and preventing cardiovascular diseases.

Phytochemicals are bioactive compounds produced naturally in plants and provide desirable health benefits in many diseases with minimal side effects [12, 13]. For many years, health practitioners have been using phytochemicals and medicinal plants to treat and manage hypertension. Several plants and phytochemicals have been previously reported for their antihypertensive effects. Nevertheless, more studies are required to clarify their effectiveness [14–16]. Monoterpenes constitute 90% of essential oils and have many biological and pharmacological properties, including antimicrobial, antihypertensive, antioxidant, antiarrhythmic, and antispasmodic activities [17, 18]. Pulegone, also known as cisisopulegone, is a natural monoterpene [19]. Pulegone has been previously reported to have numerous pharmacological effects such as cardiovascular [20], antimicrobial [21], antioxidant [22], antinociceptive [23], analgesic [24], anti-inflammatory [25], and antispasmodic activities [26].

It is found predominantly in peppermint and pennyroyal (*Mentha* spp) [27]. Other plants that contain pulegone in high quantity are *Ziziphora taurica* M. Bieb. (81.86%) [28], *Mentha spicata* L. (72.1%) [29], *Cyclotrichium niveum* (Boiss.) Manden. and Scheng. (76.84%) [30], and *Micromeria cilicica* Hausskn. ex P.H. Davis (66.55%) [31]. Several toxicity studies of pulegone have been carried out in previous studies. The subcutaneous median lethal dose (LD50) has been estimated as 1.709 mg/kg in mice, the intraperitoneal LD50 as 150 mg/kg in rats, and the intravenous LD50 as (330 mg/kg) in dogs. However, no orally administered dose of LD50 through gavage is available [32]. Acute oral toxicity studies in rats indicate that pulegone is not associated with toxicological effects at 10 mg/kg and 100 mg/kg [33]. Various medicinal plants containing pulegone as their phytoconstituents have been reported to have antihypertensive activity. *Mentha suaveolens* Ehrh. has been reported to demonstrate hypotensive effects in a noradrenaline-induced hypertensive rat model [34]. Similarly, *Mentha pulegium* L. has been reported to reduce blood pressure in L-NAME-induced hypertensive rats [35]. However, no such activity has been carried out to evaluate the effect of pulegone against hypertension in L-NAME-induced hypertensive rats. In many animal models, L-NG-nitroarginine methyl ester (L-NAME) is a NOS inhibitor that induces arterial hypertension and alters antioxidant enzymes, serum nitric oxide, lipid profile, and renal functions [36–38]. Hence, the current study evaluated pulegone's antihypertensive and vasoprotective effects in L-NAME-induced hypertensive rats and the possible mechanisms underlying this effect. The impact of pulegone on different parameters such as nitric oxide, antioxidant enzymes, lipid profile, hepatic enzymes, urine, and salt retention was also assessed in this model.

## 2. Materials and Methods

### 2.1. Chemicals

Drugs and chemicals of standard analytical grade were used in this experiment. The following drugs, acetylcholine chloride, xylazine, ketamine, phenylephrine, captopril, pulegone, N-nitro-L-arginine methyl ester (L-NAME), atropine, and indomethacin, were procured from Sigma-Aldrich (St. Louis, MA, USA).

### 2.2. In Vitro Antioxidant Assay

#### 2.2.1. DPPH Radical Scavenging Activity

DPPH solution (1,1-diphenyl-2-picryl-hydrazine) of 0.3 mM was made in ethanol. Various concentrations of pulegone ranging from 62.5 *μ*g to 500 *μ*g having 95 *μ*L of DPPH solution were mixed in ethanol. The combination was incubated at 37°C for 30 min after being disseminated in 96-well plates. The radical scavenging activity was evaluated by comparing it with the control, and absorbance was assessed by a microlitre plate system (Spectramax plus 384 Molecular Device, USA) at 515 nm. The standard used was BHA [39].

(1) $$\begin{array}{c} \text{DPPH scavenging activity }\left(\%\right)=\frac{\text{Ac}–\text{As}}{\text{Ac}\times 100}, \end{array}$$

where *Ac* means absorbance of control (DMSO treated) and *As* means absorbance of sample.

### 2.3. Acetylcholinesterase (AChE) Inhibitory Activity Assay

Acetylcholinesterase (500 U) was thoroughly mixed in Tris-HCl buffer (pH 7.8) and alleviated by adding bovine serum albumin (0.1% w/v). Pulegone was placed carefully on the TLC plates (0.01 to 1000 ng/spot). In his experiment, physostigmine was used as the control. The plates loaded with acetylcholinesterase solution (3.33 U/mL) were dried and incubated at 37°C for 20 min. Enzymatic activity was identified by spraying a mixture (0.25%) of 1-naphthyl acetate in ethanol and a (0.25%) mixture of fast blue B salt (20 mL). The inhibition of acetylcholinesterase enzyme resulted in bright regions on a purple background [40].

### 2.4. Animals

Male Wistar rats with a body weight of 200-250 g were kept at a controlled temperature (25 ± 1°C) and humidity conditions (60 ± 5%). Animals were fed with diet and water on an ad libitum basis. This experiment was performed in the light of the recommended principles for animal research and documented approval (#IREC-2020-64) sanctioned by the institutional research and ethical committee of the Faculty of Pharmacy, University of Lahore.

### 2.5. Pharmacological Studies on Pulegone

#### 2.5.1. Measuring Blood Pressure in Anesthetized Normotensive Rats

Normotensive rats were anesthetized by injecting ketamine (80 mg/kg/i.p) and xylazine (10 mg/kg; i.p) and placed on a dissevering slab beneath a lamp to maintain constant body temperature. The trachea was exposed and cannulated to promote persistent breathing. A polyethylene tube (PE 50) was positioned into the jugular vein to administer the drug. In contrast, another line was placed into the carotid artery coupled to a pressure transducer and PowerLab data acquisition system (ADI Instruments, Australia), which measured blood pressure (BP) and heart rate (HR). Consequently, the rats were stabilized for 30 minutes before further analysis. To measure the hypotensive dose-response relationship of pulegone, normotensive rats were treated with a placebo (i.v. 1% tween 80) or different doses of pulegone (1 mg/kg, 5 mg/kg, 10 mg/kg, 20 mg/kg, and 30 mg/kg). Tween 80 was administered to the control group to confirm that the detected effects were unrelated to the vehicle. After injecting pulegone, cardiovascular parameters (BP and HR) were recorded for 45 minutes [41].

#### 2.5.2. Evaluation of Possible Underlying Mechanisms of the Hypotensive Effect of Pulegone

Animals were anesthetized, and blood pressure was recorded, as mentioned earlier. The normotensive group of rats received the following antagonists (atropine: 1 mg/kg (muscarinic receptor blocker), indomethacin: 5 mg/kg (COX inhibitor), and L-NAME: 20 mg/kg (NOS inhibitor)) intravenously 10 minutes before administering pulegone. Then, blood pressure was recorded for 45 minutes using invasive blood pressure measuring technique [41]. For this experiment, we selected the dose of pulegone that produced at least 50% blood pressure lowering effect, which was 30 mg/kg (i.v.), as observed in the hypotensive dose-response relationship.

#### 2.5.3. Evaluating Preventative Effect of Pulegone in L-NAME-Induced Hypertensive Rats

Rats' weights (150-250 g) were allocated into six groups (*n* = 6). Hypertension was induced in rats by administering L-NAME (40 mg/kg) orally for 28 days. The group I assisted as control and received tween 80 (1%). Rats in group II received L-NAME (40 mg/kg) and served as disease control, whereas rats in group III received captopril (10 mg/kg) simultaneously with L-NAME (40 mg/kg). Groups IV, V, and VI rats received L-NAME (40 mg/kg) and three doses of pulegone (20 mg/kg, 40 mg/kg, and 80 mg/kg). According to their body weight, the drug was administered orally to rats daily at 8 : 00 AM. After every week, three readings of BP and heart rate were recorded using the tail-cuff method [42]. Furthermore, all rats' body weight, urine volume, and glucose levels were measured weekly.

#### 2.5.4. Effect of Pulegone on Endothelial Dysfunction in L-NAME-Induced Hypertensive Rats

After 28 days, vascular reactivity studies were carried out to check the integrity of the endothelium of the aorta. The thoracic aorta was removed from each rat and cleaned thoroughly to remove any intact tissues and fat. It was cut accurately into 3 mm long rings while protecting the endothelium. These rings were placed in the organ bath, and tension of 1 g was applied to provide isometric force to the aortic rings. The organ bath was occupied with 10 mL of Krebs solution containing 118 mM NaCl, 4.7 mM KCl, 1.18 mM MgSO4, 1.18 mM KH2PO4, 5.5 mM glucose, 2.5 mM CaCl2, and 25 mM NaHCO3. The organ bath was maintained at 37°C, pH 7.4, and gas (95% O2, 5% CO2). The system was made to equilibrate for about 1 hour. As soon as the system is stabilized, phenylephrine (10-6 M) is added to the organ bath, which causes contraction in the aorta rings. Acetylcholine (Ach; 10-9 to 10-4 mol/l) was formerly added to measure the relaxation of the aortic rings [42].

### 2.6. Measurement of Physical and Biochemical Parameters

#### 2.6.1. Estimation of Body Weight, Glucose, Urine Volume, Creatinine, and Sodium Levels

The body weight of all rats in each group was measured weekly. After withdrawing 0.1 mL of blood from the tail vein, plasma glucose levels were measured precisely using the Accu-Chek blood glucose system (Roche Diagnostics, Basel, Switzerland). Urine volume and electrolyte (sodium) concentrations were measured weekly from the urine collected after four hrs. After 28 days, blood was collected by cardiac puncture and centrifuged at 4000 g for 10 minutes. After separating the serum, creatinine was measured using a standard biochemical kit [43].

#### 2.6.2. Estimation of Lipid Profile and Hepatic Enzymes

At the end of the experiment, blood samples were collected and centrifuged at 4000 g for 10 minutes. The serum was made, and standard biochemical kits were used to measure the levels of triglycerides, high-density lipoprotein (HDL), low-density lipoprotein (LDL), and total cholesterol [44]. Aspartate transaminase (AST) and alanine transaminase (ALT) levels were also measured [45].

#### 2.6.3. Measurement of Serum Nitrite Levels

The oxidative product of nitric oxide (NO) is nitrite. Therefore, NO concentration was measured indirectly by measuring the level of nitrite. Briefly, N-(1-naphthyl) ethylenediamine and sulfanilic acid were mixed to produce the Griess reagent. 100 *μ*L of Griess reagent, 300 *μ*L of nitrite sample, and 2.6 mL of deionized water were measured into a spectrophotometer cuvette. This sample was placed in an incubator at room temperature for 30 minutes. Shortly after, a reference sample was organized by mixing 100 *μ*L of Griess reagent and 2.9 mL of deionized water. The absorbance was measured at 548 nm [41].

#### 2.6.4. Evaluation of Oxidative Stress Parameters

The levels of antioxidant enzyme superoxide dismutase (SOD) and catalase (CAT) were analyzed from the blood samples collected at the end of the experiment. Catalase (CAT) was determined by mixing 625 *μ*L of 50 mM potassium phosphate buffer, 100 *μ*L of 5.9 mM hydrogen peroxide (H2O2), and 35 *μ*L of supernatant. Changes in absorbance were observed at 240 nm, and an absorbance change of 0.01 units/min was specified as 1 unit CAT activity [46]. SOD was determined by mixing sodium pyrophosphate buffer and phenazine methosulphate. The supernatant was separated after centrifuging tissue homogenate at 1500 × g for 10 min. Then, 150 *μ*L of supernatant was added to 50 *μ*L of 186 mM of phenazine methosulfate and 600 *μ*L of 0.052 mM sodium pyrophosphate buffer (pH 7.0). 100 *μ*L of 780 *μ*M NADH was added to the above mixture to start the enzymatic activity. After 1 minute, glacial acetic acid (500 *μ*L) was added to finish the experiment. An absorbance of 560 nm represented the color intensity [46].

### 2.7. Reverse Transcription Polymerase Chain Reaction (PCR)

PCR studies were carried out by extracting mRNA from the plasma of rats and prepared using 100 *μ*L as the final volume. The specific mRNA sequences for the following primers are mentioned in Table 1 [47].

### 2.8. Statistical Analysis

The data were first analyzed for homogeneity of variance and a normal distribution. The data were analyzed using a one-way or two-way analysis of variance (ANOVA) followed by Bonferroni's post hoc test. The significance level adopted was 95% (*P* < 0.05). Statistical analyses and graphics were performed using GraphPad Prism version 9.5.0. for macOS.

## 3. Results

### 3.1. In Vitro DPPH Radical Scavenging Activity

The results showed that pulegone demonstrated adequate DPPH scavenging activity at IC 50 value ± *S*EM of 21.7 ± 0.83 when compared with butylated hydroxyl-anisole (BHA) at IC 50 value ± SEM of 44.2 ± 0.08.

### 3.2. In Vitro Acetylcholinesterase (AChE) Inhibitory Activity

The results showed that pulegone demonstrated AChE inhibitory activity at an IC 50 value of 46.6 ± 0.29 compared with physostigmine at an IC 50 value of 0.29 ± 0.23.

### 3.3. Hypotensive Dose-Response Relationship of Pulegone in Normotensive Rats

**Figure 1:**
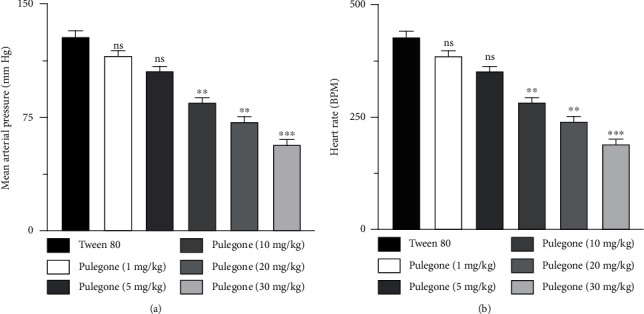
Effects of pulegone-administered i.v. on (a) mean arterial pressure (MAP) and (b) heart rate in anesthetized normotensive rats. Values are presented as mean ± SEM (*n* = 6) when one-way ANOVA was applied, followed by Bonferroni's multiple comparison test. ^∗∗∗^*P* < 0.001 and ^∗∗^*P* < 0.01 vs. vehicle-treated rats.

In this study, no substantial change in blood pressure and heart rate was observed in the rats receiving only 1% tween 80 (placebo), whereas treatment with pulegone at different doses (1 mg/kg, 5 mg/kg, 10 mg/kg, 20 mg/kg, and 30 mg/kg; i.v.) reduced mean arterial pressure (MAP) and heart rate (HR) in normotensive rats with most prominent effect at 30 mg/kg, as shown in Figure 1.

### 3.4. Pulegone Produces a Hypotensive Effect by Muscarinic Receptors and Cyclooxygenase Pathway

**Figure 2:**
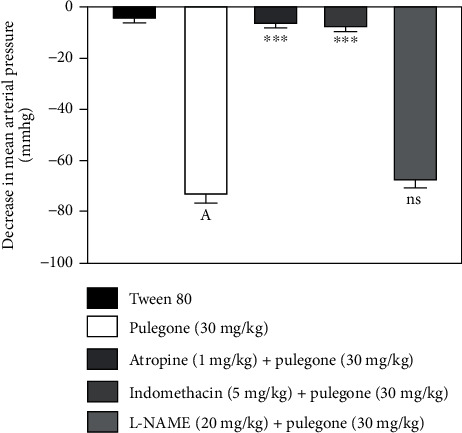
Effect of pulegone on the decrease in MAP in normotensive rats pretreated with atropine (1 mg/kg), indomethacin (5 mg/kg), and L-NAME (20 mg/kg). Values are presented as mean ± SEM (*n* = 6) when one-way ANOVA was applied, followed by Bonferroni's multiple comparison test. ^∗∗∗^*P* < 0.001 vs. pulegone. (a) *P* < 0.001 vs. vehicle-treated rats.

Treatment with pulegone produced a hypotensive effect in normotensive rats that were significantly prevented with atropine and indomethacin. Treatment with only pulegone (30 mg/kg) caused a 77.43 mm Hg decrease in MAP, whereas, in the presence of atropine and indomethacin, pulegone reduced MAP to 5.42. and 7.58 mm Hg, respectively. However, pretreatment with L-NAME (40 mg/kg) did not change the hypotensive effect of pulegone, as shown in Figure 2.

### 3.5. Evaluation of the Preventive Effect of Pulegone in L-NAME-Induced Hypertensive Rat Model

#### 3.5.1. Effect of Pulegone on Mean Arterial Pressure and Heart Rate in L-NAME-Induced Hypertensive Rats

**Figure 3:**
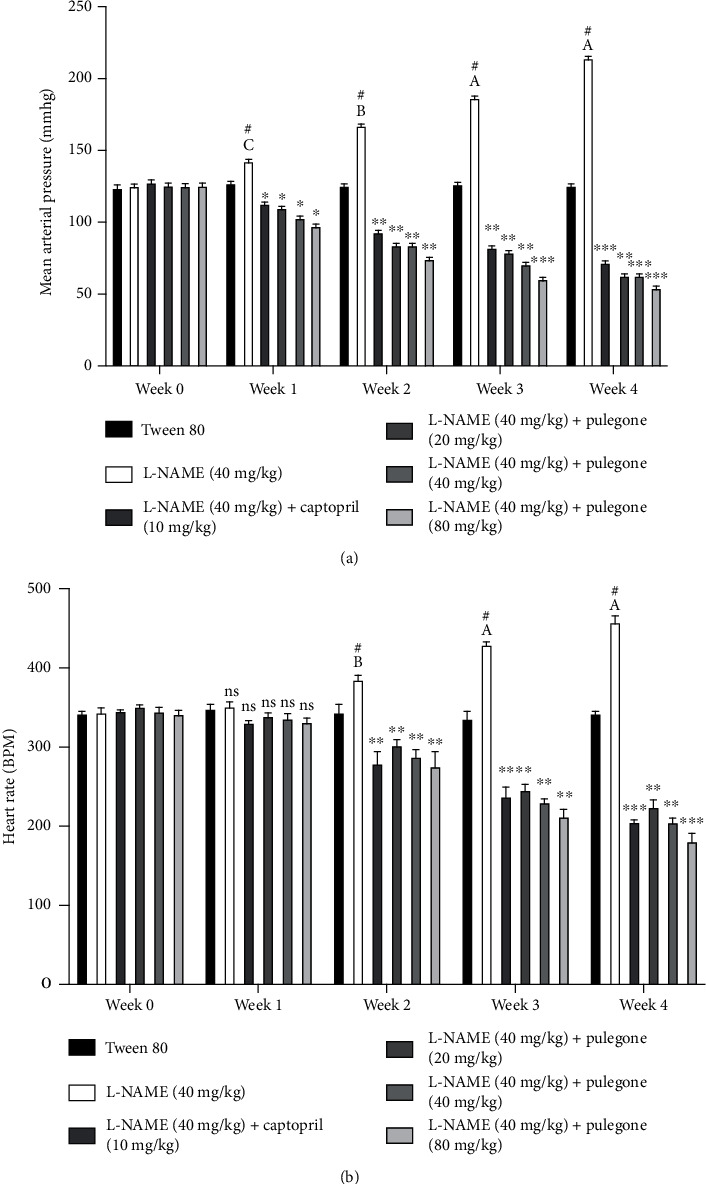
Effect of pulegone on (a) mean arterial pressure and (b) heart rate in L-NAME-induced hypertensive rats. Values are presented as mean ± SEM (*n* = 6). Two-way ANOVA showed a significant interaction between the variables, and Bonferroni's multiple comparison tests were performed. ^∗∗∗^*P* < 0.001, ^∗∗^*P* < 0.01, and ^∗^*P* < 0.05 vs. L-NAME-treated rats. ^∗^(A) *P* < 0.001, (B) *P* < 0.01, and (C) *P* < 0.05 vs. vehicle-treated rats. # denotes *P* < 0.05 compared to the previous week.

Administration of L-NAME (40 mg/kg) resulted in a significant increase in MAP compared to placebo-treated rats, seen from week 2 of treatment. Treatment with captopril prevented increased BP and heart rate due to L-NAME administration. Interestingly, pulegone at doses of 20 mg/kg, 40 mg/kg, and 80 mg/kg prevented the increase in MAP and heart rate, and this effect continued throughout week three and week four, as shown in Figure 3. At the end of week 4, L-NAME rats showed significant increases in MAP and HR compared with vehicle-treated rats.

#### 3.5.2. Effect of Pulegone on Vascular Dysfunction in L-NAME-Induced Hypertensive Rats

**Figure 4:**
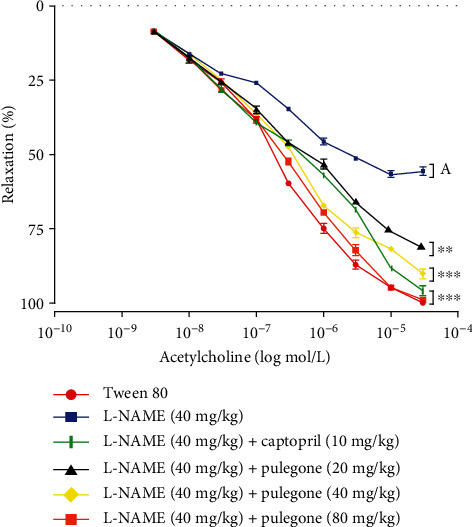
Effect of pulegone on vascular dysfunction in L-NAME-induced hypertensive rats. Values are expressed as mean ± SEM (*n* = 6) when one-way ANOVA was applied, followed by Bonferroni's multiple comparison test. ^∗∗∗^*P* < 0.001 and ^∗∗^*P* < 0.01 vs. L-NAME-treated rats. (a) *P* < 0.001 vs. vehicle-treated rats.

Our current study showed that L-NAME impairs vascular response to acetylcholine in phenylephrine-precontracted aortic rings by inhibiting vasorelaxation in the aortic rings. Treatment with captopril (10 mg/kg) and pulegone (20 mg/kg, 40 mg/kg, and 80 mg/kg) improved vascular response to acetylcholine by exhibiting endothelium-dependent vasorelaxation in L-NAME-pretreated rats. Results are illustrated in Figure 4.

### 3.6. Effect of Pulegone on Physical and Biochemical Parameters

#### 3.6.1. Effect of Pulegone on Body Weight and Glucose in L-NAME-Induced Hypertensive Rats

Compared to normal rats, L-NAME hypertensive rats demonstrated raised glucose levels and a marked reduction in body weight in 4 weeks of experimental design. Treatment with pulegone (20 mg/kg, 40 mg/kg, and 80 mg/kg) reduced body weight and glucose levels consecutively compared to L-NAME-induced hypertensive rats. Treatment with captopril increased body weight in L-NAME-pretreated rats (Table 2).

#### 3.6.2. Effect of Pulegone on Serum Nitrite in L-NAME-Induced Hypertensive Rats

**Figure 5:**
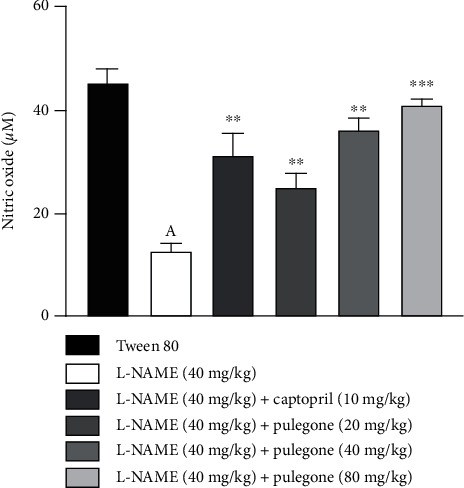
Effect of pulegone on nitric oxide (NO) in L-NAME-induced hypertensive rats. Values are expressed as mean ± SEM (*n* = 6) when one-way ANOVA was applied, followed by Bonferroni's multiple comparison test. ^∗∗∗^*P* < 0.001 and ^∗∗^*P* < 0.01 vs. L-NAME-treated rats. (a) *P* < 0.001 vs. vehicle-treated rats.

Serum nitrite levels from diverse groups are shown in Figure 5. In rats treated with L-NAME, nitric oxide levels decreased significantly compared to the vehicle-treated rats based on the inhibition of nitric oxide synthase by L-NAME. Treatment with pulegone (20 mg/kg, 40 mg/kg, and 80 mg/kg) and captopril (10 mg/kg) restored NO levels to normal in L-NAME-pretreated rats.

#### 3.6.3. Effect of Pulegone on Lipid Profile in L-NAME-Induced Hypertensive Rats

**Figure 6:**
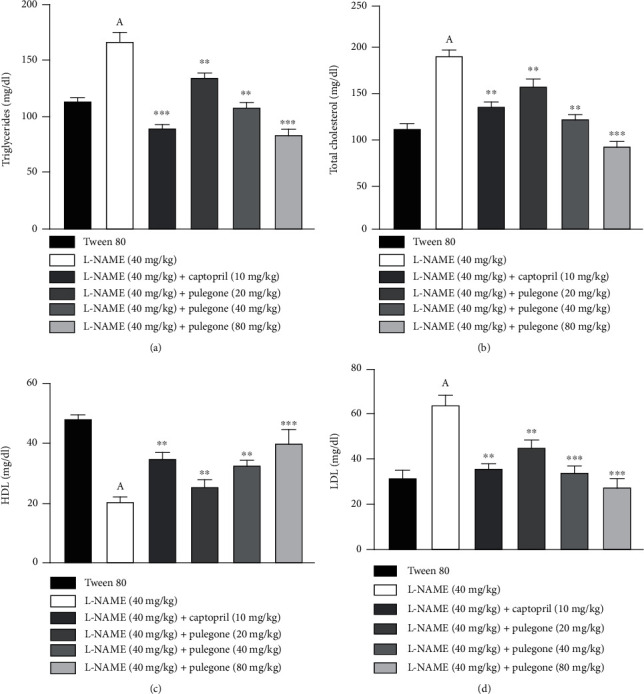
Effect of pulegone on (a) triglycerides, (b) total cholesterol, (c) HDL, and (d) LDL in L-NAME-induced hypertensive rats. Values are expressed as mean ± SEM (*n* = 6) when one-way ANOVA was applied, followed by Bonferroni's multiple comparison test. ^∗∗∗^*P* < 0.001 and ^∗∗^*P* < 0.01 vs. L-NAME-treated rats. (A) *P* < 0.001 vs. vehicle-treated rats.

The results of this experiment indicated dyslipidemia in L-NAME-treated rats and demonstrated by raised triglycerides, cholesterol, and LDL combined with decreased levels of HDL as compared to the vehicle-treated group. Simultaneous administration of pulegone (20 mg/kg, 40 mg/kg, and 80 mg/kg) restored the concentration of triglycerides, total cholesterol, LDL, and HDL. Treatment with captopril in L-NAME-pretreated rats caused a decrease in total cholesterol and triglycerides and increased HDL levels (Figure 6).

#### 3.6.4. Effect of Pulegone on Endogenous Antioxidant Enzymes in L-NAME-Induced Hypertensive Rats

**Figure 7:**
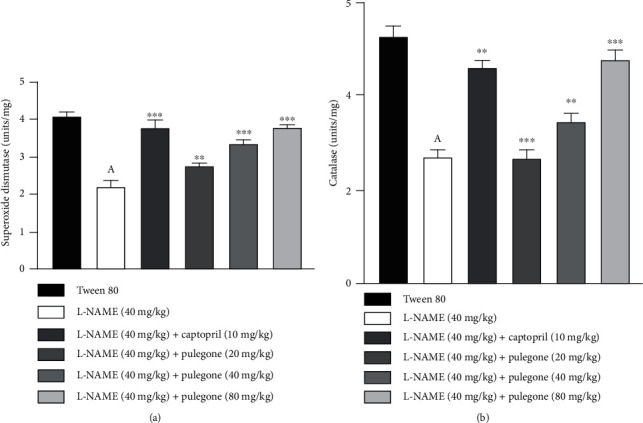
Effect of pulegone on (a) SOD and (b) CAT in L-NAME-induced hypertensive rats. Values are presented as mean ± SEM (*n* = 6) when one-way ANOVA was applied, followed by Bonferroni's multiple comparison test. ^∗∗∗^*P* < 0.001 and ^∗∗^*P* < 0.01 vs. L-NAME-treated rats. (A) *P* < 0.001 vs. vehicle-treated rats.

Administration of L-NAME for four weeks resulted in a reduction of endogenous antioxidant enzymes as compared to vehicle-treated rats. However, treatment with pulegone significantly restored plasma SOD and CAT levels with more pronounced effects at 40 mg/kg and 80 mg/kg (Figure 7). Treatment with captopril prevented the decrease in SOD and CAT compared to the hypertensive rats.

#### 3.6.5. Effect of Pulegone on Liver Enzymes in L-NAME-Induced Hypertensive Rats

**Figure 8:**
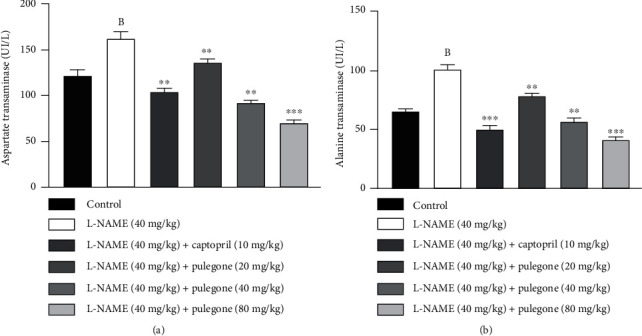
Effect of pulegone on (a) AST and (b) ALT in L-NAME-induced hypertensive rats. Values are presented as mean ± SEM (*n* = 6) when one-way ANOVA was applied, followed by Bonferroni's multiple comparison test. ^∗∗∗^*P* < 0.001 and ^∗∗^*P* < 0.01 vs. L-NAME-treated rats. (B) *P* < 0.01 vs. vehicle-treated rats.

In our present study, liver enzymes (AST and ALT) were favorably raised after administering L-NAME compared to vehicle-treated rats, indicating liver damage (Figure 8). Treatment with pulegone restored augmented levels of AST and ALT, with maximum effects at 80 mg/kg compared to the L-NAME group. Additionally, treatment with captopril reinstated AST and ALT levels in L-NAME-pretreated rats.

#### 3.6.6. Effect of Pulegone on Serum Creatinine, Urine Volume, and Sodium Levels in L-NAME-Induced Hypertensive Rats

**Figure 9:**
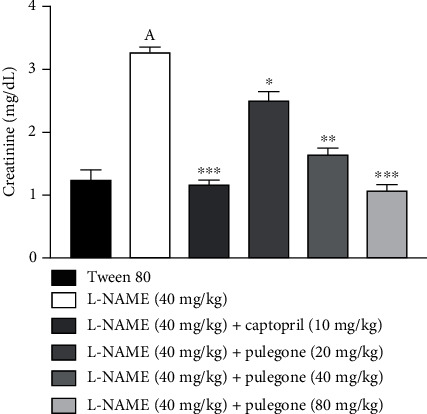
Effect of pulegone on serum creatinine in L-NAME-induced hypertensive rats. Values are presented as mean ± SEM (*n* = 6) when one-way ANOVA was applied, followed by Bonferroni's multiple comparison test. ^∗∗∗^*P* < 0.001, ^∗∗^*P* < 0.01, and ^∗^*P* < 0.05 vs. L-NAME-treated rats. (a) *P* < 0.001 vs. vehicle-treated rats.

In this experiment, serum creatinine levels were increased, and urine sodium levels were decreased in pretreated L-NAME rats (Figure 9). Nevertheless, treatment with pulegone at 20 mg/kg, 40 mg/kg, 80 mg/kg, and captopril (10 mg/kg) restored creatinine, urine sodium, and urine volume (Table 3) basal values as compared to hypertensive rats.

### 3.7. Real-Time Quantitative Polymerase Chain Reaction (qPCR)

#### 3.7.1. Effects of Pulegone on mRNA Expression of eNOS (Nitric Oxide Synthase), ACE (Angiotensin-Converting Enzyme), ICAM1 (Intracellular Adhesion Molecule 1), and EDN1 (Endothelin-1) in L-NAME-Induced Hypertensive Rats

**Figure 10:**
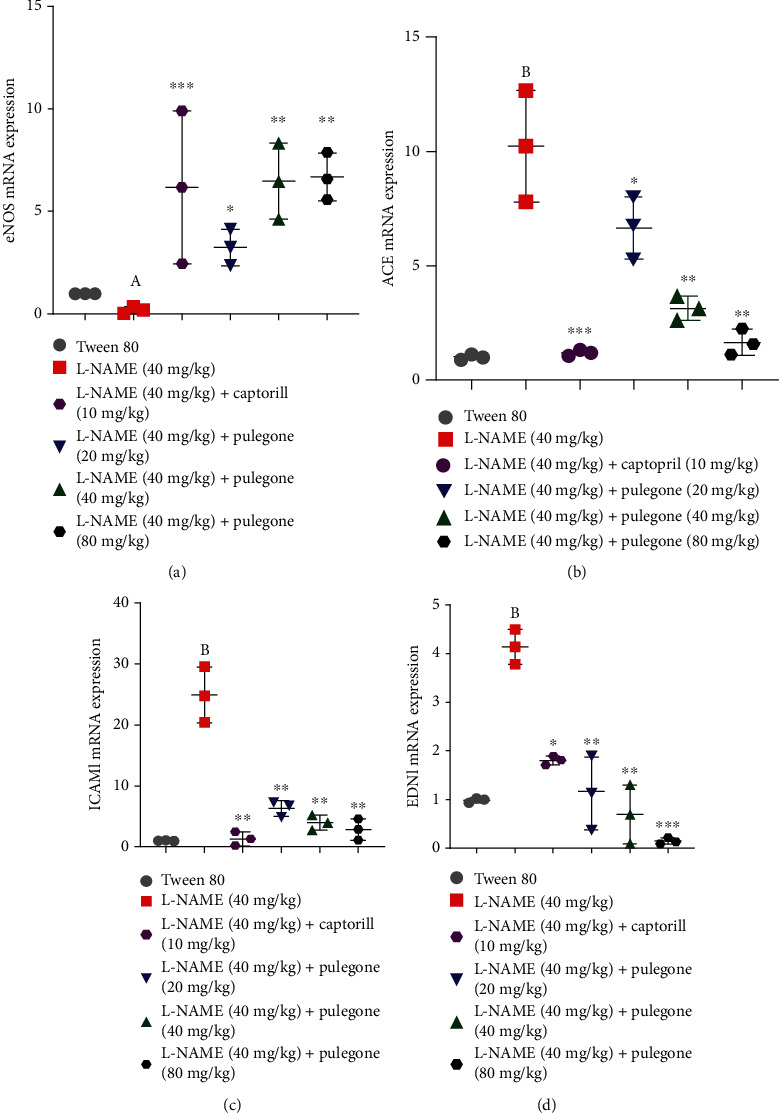
Expression of mRNA for (a) eNOS, (b) ACE, (c) ICAM1, and (d) EDN1 analyzed by RT-PCR in L-NAME-induced hypertensive rats. Values are expressed as mean ± SEM (*n* = 6) when one-way ANOVA was applied, followed by Bonferroni's multiple comparison test. ^∗∗∗^*P* < 0.001, ^∗∗^*P* < 0.01, and ^∗^*P* < 0.05 vs. L-NAME-treated rats. (A) *P* < 0.005 and (B) *P* < 0.001 vs. vehicle-treated rats.

In this study, eNOS mRNA expression is reduced in pretreated L-NAME rats compared to vehicle-treated rats (Figure 10(a)). The simultaneous treatment with pulegone (40 mg/kg and 80 mg/kg) raised the mRNA expression of eNOS in hypertensive rats. Moreover, the diminished expression of eNOS was also reversed by administering captopril (10 mg/kg) in L-NAME-treated rats. Furthermore, mRNA expression of ACE, ICAM1, and EDN1 was significantly increased in L-NAME-treated rats when compared with the vehicle-treated group (Figures 10(b)–10(d)). Continuous treatment with different doses of pulegone (20 mg/kg, 40 mg/kg, and 80 mg/kg) markedly reduced mRNA expression of ACE, ICAM1, and EDN1 in hypertensive rats. Similarly, our standard drug captopril also suppressed the mRNA expression of ACE, ICAM1, and EDN1 towards average values in L-NAME-pretreated rats.

## 4. Discussion

Natural products and phytochemicals derived from medicinal plants have led to new drugs with desirable health benefits and fewer side effects [12, 48]. Pulegone, a monoterpene, has been stated previously to have numerous pharmacological activities such as cardiovascular [20], antioxidant [22], analgesic [24], and anti-inflammatory activities [25]. Our present study evaluated pulegone for its hypotensive activity in normotensive rats and antihypertensive activity in the L-NAME-induced hypertensive rat model. Pulegone prevented L-NAME-induced hypertension by demonstrating a hypotensive effect by activating muscarinic receptors and the cyclooxygenase pathway. It also prevented oxidative stress, vascular dysfunction, and urine and sodium retention in L-NAME-treated rats. To measure the hypotensive dose-response relationship of pulegone, normotensive rats were administered a placebo (1% tween 80) and different doses of pulegone (1 mg/kg, 5 mg/kg, 10 mg/kg, 20 mg/kg, and 30 mg/kg; i.v.). Pulegone showed a dose-dependent hypotensive effect in anesthetized normotensive rats, with the highest impact at 30 mg/kg. Data obtained with a mechanism-based study indicates that the hypotensive effect of pulegone was reduced in the presence of atropine and indomethacin. In contrast, L-NAME did not change its hypotensive effect. Indomethacin, a potent inhibitor of COX-1 and COX-2, inhibits the synthesis of prostaglandins. Previous literature reported that prostaglandins such as prostacyclin (PGI2) reduce blood pressure by increasing the level of cAMP leading to vasorelaxation and, ultimately, hypotension [8, 9]. As in our study, indomethacin blocked the hypotensive effects of pulegone in rats, indicating that COX pathways may be involved in the hypotensive effect seen with pulegone.

Atropine is a muscarinic receptor blocker that blocks the inhibitory effects of acetylcholine [6]. Previous observations show that acetylcholine (via M3 receptors) enhances NO release [7]. It is also known that M2 muscarinic receptors, via inhibition of adenylyl cyclase, cause indirect contraction of bladder smooth muscles, whereas M3 receptors directly mediate contractile effects of acetylcholine in the detrusor muscle, leading to enhanced urination [41]. As in our study, the presence of atropine blocked pulegone's effects of pulegone, so it is hypothesized that pulegone may act through M3 receptors on detrusor muscle which causes its contraction and enhances diuresis, therefore reducing blood pressure. Increased urine and sodium excretion in pretreated L-NAME rats with pulegone also support the activation of this pathway. Moreover, pulegone also demonstrated AChE inhibitory activity. Therefore, it can be hypothesized that pulegone enhances NO production, stimulates muscarinic receptors, and increases urine flow to produce a hypotensive effect. Our results were from a previous study where *Cyperus esculentus* L. inhibits AChE activity and augments the release of NO from endothelial cells [49].

In this study, pulegone significantly prevented hypertension in the L-NAME-induced hypertensive rat model. While evaluating its preventative effect, we used three different doses of pulegone (20 mg/kg, 40 mg/kg, and 80 mg/kg) with previous reference to the fact that these doses were safe, and no toxic activity is observed [33]. The primary effect of parasympathetic stimulation is to decrease cardiac output by decreasing heart rate. Activation of M₂ receptors on cardiac muscle inhibits adenyl cyclase and increases potassium conductance which reduces heart rate [50]. So, it can be hypothesized that pulegone decreases heart rate through the above mechanism. Administration of pulegone in pretreated L-NAME rats decreases body weight and glucose levels. Based on previous studies, a decrease in water and food consumption reduces body weight. Moreover, as our drug demonstrated hypolipidemic properties, this further reduces body weight [51]. Decreased NO bioavailability increases levels of glucose and leads to diabetes mellitus. Our drug pulegone increases NO levels which decrease glucose levels which are primarily mediated by the sGC–cGMP pathway [52].

It is well-known that the administration of L-NAME inhibits eNOS synthase, decreases NO bioavailability, and increases blood pressure. Various studies have supported that L-NAME reduces NO/cGMP activity, activates the renin system, and increases sympathetic tone, raising blood pressure [44, 53]. Endothelial cells synthesize and release nitric oxide, which is involved in vascular cell proliferation [54]. Administration of L-NAME in our current study reduces NO levels, which causes endothelial dysfunction and hypertension [55]. This outcome was similar to former studies, where L-NAME increased BP and significantly decreased serum NO by inhibiting the enzyme NO synthase [46]. Treatment with pulegone prevented the decrease in plasma NO levels. Previous studies have proven that many phytoconstituents increased NO levels by activating eNOS [54]. Therefore, an increased level of NO in rats treated with pulegone could be due to an increase in the activity of eNOS. This study's results align with Aekthammarat et al. [56], who reported that *Moringa oleifera* Lam. improves vascular function by enhancing NO production. Our results also showed that L-NAME impairs vascular response to acetylcholine by inhibiting vasorelaxation in the aortic rings. This was relevant in a previous study where Ajebli and Eddouks [35] reported that L-NAME attenuated the vasorelaxant effect. This outcome may result from NO deficiency, which increases vasoconstrictors derived from the cyclooxygenase pathway [57]. Thus, it can be postulated that pulegone prevented endothelial dysfunction and hypertension in L-NAME-treated rats by preventing the decrease in NO level.

Oxidative stress and ROS production lead to the development of hypertension in L-NAME-treated rats [58]. Supporting evidence shows that oxidative stress plays a vital role in hypertensive rats by reducing endogenous antioxidant enzymes [46]. Oxidative stress reduces NO production, impairs vasodilation, and causes endothelial dysfunction [59]. In our study, levels of SOD and CAT were prominently reduced in L-NAME-treated rats. Our results reveal that induction with L-NAME increases ROS and decreases antioxidant enzymes. However, treatment with pulegone was able to mitigate oxidative stress by restoring SOD and CAT activities in pretreated L-NAME rats. This finding is from a recent study that showed that carvone significantly increased SOD and CAT levels by decreasing ROS production in hypertensive rats [60]. The above results suggest the favorable effects of pulegone in hypertension by augmenting endogenous antioxidants and enhancing NO production. To confirm the impact of pulegone against oxidative stress, DPPH radical scavenging assay was performed, which indicated the antioxidant activity of pulegone due to its hydrogen ion-donating ability [61].

Previous studies reported that inhibition of NO synthase by L-NAME disturbs levels of HDL, LDL, total cholesterol, and triglycerides which causes hypertension [45]. Our results indicate dyslipidemia in L-NAME-treated rats, which is proven by elevated triglycerides, cholesterol, and LDL coupled with reduced HDL levels. The augmented levels of triglyceride and cholesterol might be due to a reduction in the oxidation of fatty acids [62]. Captopril and pulegone modified the lipid profile by restoring TC, LDL, TG, and HDL levels. A recent study by Abdel-Rahman et al. [44] supported the above finding, where *Olea europaea* L. demonstrates significant hypolipidemic activity in hypertensive rats. These outcomes clearly show pulegone's hypolipidemic properties, which may contribute to its hypotensive effect.

It is evidenced that impairment of AST and ALT raises blood pressure. Liver enzymes such as ALT and AST are elevated in plasma and used as markers of hepatic dysfunction in cardiovascular diseases such as hypertension [63]. In our present study, AST and ALT were amplified in L-NAME-treated rats. L-NAME increases oxidative stress and causes liver damage [45]. Treatment with pulegone exhibited a protective effect on the liver by reducing AST and ALT levels. A previous study reported that lutein provided hepatic protection by decreasing AST and ALT levels in L-NAME-treated rats [59].

Kidneys maintain water and electrolytes in the human body with the help of NO, which regulates the renal system [64]. In our present study, urine volume and sodium levels were significantly reduced, whereas creatinine levels were considerably increased in the serum of L-NAME-treated rats. This study implies previous findings where RAS activation and inhibition of NO by L-NAME lead to decreased sodium excretion and hypertension [45, 65]. Treatment with pulegone in L-NAME-treated rats significantly increased the urine volume and sodium excretion, reducing blood pressure. Therefore, it is hypothesized that pulegone acts through M2/M3 receptors (urinary bladder muscles), enhancing diuresis, increasing NO levels, and lowering blood pressure [7, 41].

To obtain more information about how pulegone reduces blood pressure, mRNA expression of eNOS, ACE, ICAM1, and EDN1 was measured. In our current study, administration of L-NAME increases mRNA expression of ACE, ICAM1, and EDN1, and mRNA expression of eNOS was significantly reduced due to the inhibition of eNOS. L-NAME activates ACE and increases ANG II production by causing vascular inflammation and atherosclerosis, contributing to hypertension [66, 67]. However, treatment with pulegone suppressed ACE expression. This was relevant to our previous studies, which reported that the *Sorbus commixta* Hedl. demonstrated a hypotensive effect by downregulating mRNA expression of ACE in L-NAME-treated rats [68].

Furthermore, the administration of L-NAME raises ICAM-1 level by inhibiting eNOS and T-cell infiltration [69, 70]. T-cell infiltration causes ANG II production, which is a potent vasoconstrictor, and thus increases blood pressure. Former data reveals that plant extracts have suppressed ICAM1 levels in hypertensive rats via NO/cGMP signaling [71, 72]. Therefore, the anti-inflammatory response of pulegone could be facilitated by restoring NO levels and reducing the infiltration of immune cells. Moreover, L-NAME increases EDN1 expression by stimulating ROS, which induces endothelial dysfunction and hypertension [68, 73]. Treatment with pulegone reduces augmented EDN1 expression in pretreated L-NAME rats. Therefore, it may be supposed that pulegone restores high expression of EDN1 by reducing oxidative stress and preventing vascular hypertrophy.

## 5. Conclusion

In conclusion, our results confirm that pulegone prevents L-NAME-induced hypertension by demonstrating a hypotensive effect through muscarinic receptors and the cyclooxygenase pathway. Pulegone improved liver and kidney functions and lipid profile and restored antioxidant enzymes in L-NAME-treated rats. It also prevented vascular dysfunction by restoring NO levels in endothelial cells. Moreover, pulegone restored mRNA expression of eNOS, ACE, ICAM1, and EDN1. These findings indicate that pulegone can be a potential future candidate in the management of hypertension.

## Figures and Tables

**Table 1:** Primers used in PCR.

Markers	Primers
Endothelial nitric oxide synthase (eNOS)	Forward: 5′-CATGTTTGTCTGCGGTGATGT-3′
	Reverse: 5′-GCTTCATCCAGCTCCATGCT-3′
Angiotensin-converting enzyme (ACE)	Forward: 5′-GCAGTACAAAGACTTGCCTG-3′
	Reverse: 5′-TGGCAGAGGCTGACATGTTA-3′
Intercellular adhesion molecule 1 (ICAM1)	Forward:5′-TTTCGATCTTCCGACTAGGG-3′
	Reverse: 5′-AGCTTCAGAGGCAGGAAACA-3′
Endothelin 1 (EDN1)	Forward: 5′-ATGGATTATTTTCCCGTGAT-3′
	Reverse: 5′-GGGAGTGTTGACCCAGATGA-3′

**Table 2:** Effect of pulegone on body weight and glucose in L-NAME-induced hypertensive rats.

Treatment	Body weight (g)					Glucose (mg/dL)				
Weeks	Week 0	Week 1	Week 2	Week 3	Week 4	Week 0	Week 1	Week 2	Week 3	Week 4
Tween 80	218 ± 4.41	226 ± 0.88	236 ± 0.51	240 ± 2.88	245 ± 1.22	87.0 ± 4.61	92.3 ± 1.20	98.2 ± 0.54	103 ± 1.76	110 ± 0.77
L-NAME (40 mg/kg)	244 ± 3.48	237 ± 0.55^ns^	218 ± 2.02^b^	209 ± 1.20^b^	202 ± 0.65^a^	89.3 ± 4.05	97.3 ± 0.66^ns^	114 ± 2.60^b^	131 ± 2.08^b^	140 ± 0.50^a^
L-NAME (40 mg/kg) and captopril (10 mg/kg)	210 ± 6.65	219 ± 0.54^ns^	232 ± 1.15^∗∗^	246 ± 2.40^∗∗∗^	253 ± 1.88^∗∗∗^	102 ± 5.04	98.0 ± 0.577^ns^	89.0 ± 0.57^∗∗^	76.3 ± 0.88^∗∗∗^	70.5 ± 1.33^∗∗∗^
L-NAME (40 mg/kg) and pulegone (20 mg/kg)	219 ± 4.72	213 ± 0.88^ns^	207 ± 3.51^∗∗^	191 ± 4.41^∗∗∗^	185 ± 2.76^∗∗∗^	92.6 ± 7.68	89.3 ± 0.33^ns^	83.3 ± 0.88^∗∗^	77.6 ± 1.45^∗∗∗^	73.2 ± 0.11^∗∗∗^
L-NAME (40 mg/kg) and pulegone (40 mg/kg)	241 ± 6.64	229 ± 2.56^ns^	210 ± 2.96^∗^	191 ± 5.00^∗∗^	182 ± 3.26^∗∗^	87.6 ± 6.03	84.3 ± 0.34^ns^	78.6 ± 0.882^∗∗∗^	71.3 ± 0.66^∗∗∗^	67.7 ± 0.61^∗∗∗^
L-NAME (40 mg/kg) and pulegone (80 mg/kg)	221 ± 9.28	215 ± 0.86^ns^	196 ± 2.30^∗∗^	189 ± 6.22^∗∗^	176 ± 4.55^∗∗∗^	94.3 ± 6.64	91.3 ± 0.35^ns^	84.1 ± 0.54^∗∗^	73.6 ± 1.20^∗∗∗^	69.7 ± 0.64^∗∗∗^

The results are stated as mean ± SEM (*n* = 6), where one-way ANOVA was applied, followed by Dunnett's multiple comparison test: ^∗∗∗^*P* < 0.001, ^∗∗^*P* < 0.01, ^∗^*P* < 0.05 vs. L-NAME-treated rats. ^a^*P* < 0.001 and ^b^*P* < 0.01 vs. vehicle-treated rats.

**Table 3:** Effect of pulegone on urine volume and sodium level in L-NAME-induced hypertensive rats.

Treatment (mg/kg)	Sodium (mmol/l)					Urine volume (ml/kg) (after 4 hr)				
Weeks	Week 0	Week 1	Week 2	Week 3	Week 4	Week 0	Week 1	Week 2	Week 3	Week 4
Tween 80	120 ± 1.73	125 ± 3.78	128 ± 1.66	135 ± 1.28	137 ± 4.65	2.3 ± 0.33	2.6 ± 0.53	3.0 ± 0.34	3.2 ± 0.78	3.5 ± 0.79
L-NAME (40 mg/kg)	113 ± 0.57	109 ± 6.83^ns^	95.6 ± 1.21^b^	80.7 ± 4.4^a^	70.2 ± 3.29^a^	2.5 ± 0.41	2.2 ± 1.32^ns^	1.5 ± 1.11^b^	1.0 ± 0.69^a^	0.6 ± 2.55^a^
L-NAME (40 mg/kg) and captopril (10 mg/kg)	100 ± 1.78	110 ± 4.88^ns^	125 ± 5.83^∗∗^	132 ± 7.55^∗∗∗^	139 ± 3.28^∗∗∗^	2.2 ± 0.77	2.4 ± 0.55^ns^	3.1 ± 0.84^∗∗^	3.8 ± 1.98^∗∗∗^	4.5 ± 0.45^∗∗∗^
L-NAME (40 mg/kg) and pulegone (20 mg/kg)	117 ± 2.73	121 ± 1.22^∗^	129 ± 7.33^∗∗∗^	135 ± 4.89^∗∗∗^	140 ± 4.63^∗∗∗^	2.5 ± 1.33	2.7 ± 0.42^ns^	3.7 ± 0.21^∗∗^	4.5 ± 0.71^∗∗∗^	5.7 ± 0.66^∗∗∗^
L-NAME (40 mg/kg) and pulegone (40 mg/kg)	110 ± 2.48	118 ± 5.77^∗^	127 ± 1.76^∗∗∗^	141 ± 5.44^∗∗∗^	152 ± 3.79^∗∗∗^	2.5 ± 0.27	2.7 ± 0.33^ns^	3.8 ± 1.31^∗∗^	4.9 ± 1.67^∗∗∗^	6.2 ± 0.68^∗∗∗^
L-NAME (40 mg/kg) and pulegone (80 mg/kg)	108 ± 3.49	115 ± 4.57^∗^	130 ± 1.33^∗∗∗^	145 ± 4.38^∗∗∗^	158 ± 1.65^∗∗∗^	2.3 ± 1.38	2.7 ± 0.52^ns^	3.8 ± 0.82^∗∗^	5.2 ± 0.75^∗∗∗^	6.5 ± 0.40^∗∗∗^

The results are stated as mean ± SEM (*n* = 6), where one-way ANOVA was applied, followed by Dunnett's multiple comparison test. ^∗∗∗^*P* < 0.001, ^∗∗^*P* < 0.01, and ^∗^*P* < 0.05 vs. L-NAME-treated rats. ^a^*P* < 0.001 and ^b^*P* < 0.01 vs. vehicle-treated rats.

## Data Availability

The data used to support the findings of this study are available from the corresponding author upon request.
